# Barriers to the Broader Use of Contrast-Enhanced Ultrasound in Saudi Healthcare and Medical Research

**DOI:** 10.1055/a-2899-0967

**Published:** 2026-07-13

**Authors:** Shatha Jamal Almushayt

**Affiliations:** 1 Department of Radiological Sciences, College of Health and Rehabilitation Sciences 112893 Princess Nourah bint Abdulrahman UniversityRiyadhSaudi Arabia

**Keywords:** barriers, contrast-enhanced ultrasound, contrast agents, sonography, microbubbles

## Abstract

**Purpose** The aim of this study was to describe the current use of contrast-enhanced ultrasound in Saudi Arabia, identify barriers to its adoption, and propose strategies to support wider clinical utilization.

**Materials and methods** A cross-sectional online survey was distributed to more than 5,000 healthcare practitioners between July and October 2025. Ninety-two eligible responses were included and analyzed using descriptive statistics. An additional qualitative input was obtained from contrast agent suppliers to contextualize survey findings.

**Results** Clinical use of contrast-enhanced ultrasound was limited, with 79% of respondents reporting no current application. Among active users, most performed one to five contrast-enhanced ultrasound examinations per month. Formal contrast-enhanced ultrasound training was uncommon, despite strong interest in education. Major barriers included lack of training opportunities, low clinical awareness, regulatory constraints, and limited availability of contrast-enhanced ultrasound-capable equipment. Supplier feedback corroborated these barriers and highlighted the need for structured education and research collaboration.

**Conclusions** Contrast-enhanced ultrasound is markedly underutilized in Saudi Arabia, primarily due to educational, regulatory, and resource-related barriers. These findings support national initiatives to expand access, enhance training programs, and promote integration of contrast-enhanced ultrasound into routine clinical and research practice.

## Introduction

Contrast-enhanced ultrasound (CEUS) is an emerging imaging modality increasingly recognized for its clinical utility across various medical applications. 1​ It employs microbubble-based contrast agents (CAs) that enhance the vascular compartment, differing fundamentally from the agents used in computed tomography (CT) and magnetic resonance imaging (MRI). CEUS offers significant advantages, including a favorable safety profile; microbubbles are rapidly eliminated through the lungs and do not require renal or hepatic clearance, thereby avoiding nephrotoxicity and the need for pre-procedural laboratory testing. CEUS has diverse clinical applications across multiple specialties.

CEUS is indicated whenever the enhanced evaluation of vascularity and tissue perfusion is required beyond conventional ultrasound, particularly for accurate lesion detection and characterization, differentiation of benign and malignant pathology, and assessment of microvascular architecture. 2​ ​3​ Quantitative assessment enables the objective comparison of imaging techniques, investigation of new CA applications, characterization of tissue and tumor enhancement, assessment of therapeutic response, and reduction of variability in clinical diagnosis. Dynamic CEUS can be performed via either intravenous (IV) bolus injection or continuous infusion of the CA, with bolus injection remaining the predominant method for evaluating tumor perfusion. 4​ 5​ 6​ ​7​

CEUS is well established in liver imaging, providing real-time characterization of focal liver lesions, differentiation of benign and malignant portal vein thrombosis, guidance for biopsy and ablation procedures, and monitoring treatment responses in hepatocellular carcinoma. Its high temporal resolution and superior signal-to-background ratio enhance the detection of small lesions and real-time perfusion assessment, offering advantages over CT and MRI, while also contributing to trauma evaluation, surgical planning, and transplant assessment. 2​ ​8​ In renal and urinary applications, CEUS allows the safe evaluation of renal parenchymal perfusion, acute pyelonephritis, renal infarction, cystic and solid lesions, vesicoureteral reflux in pediatric populations, and transplant graft function, supporting the detection of vascular complications and guidance of interventional procedures. 3​ 9​​ 10​ ​11​ CEUS also contributes to bladder cancer staging, differentiating clots or hematomas from malignant lesions, assessing tumor invasion depth and grade, and providing accurate post-treatment follow-up. 12​ Intra-scrotal applications include evaluation of trauma, torsion, infections, and benign or malignant neoplasms, aiding lesion characterization and surgical planning. 3​ ​13​ In the prostate, CEUS facilitates adjunctive imaging, including perfusion assessment and targeted biopsy guidance. 3​ ​11​

CEUS enhances the evaluation of thyroid nodules by characterizing vascular perfusion and hemodynamics, improving differentiation of benign and malignant lesions, guiding ablation procedures, and providing accurate post-treatment follow-up, although standardization remains necessary. 3​ ​14​ Pancreatic applications include characterization of solid and cystic lesions, assessment of vascularity, and interventional guidance. 3​ Gallbladder imaging benefits from CEUS through the differentiation of benign and malignant wall thickening, detection of gangrenous cholecystitis, and identification of vascular complications, particularly in high-risk or emergency scenarios. 3​ ​15​ CEUS also provides the dynamic assessment of splenic and bowel perfusion, enabling the detection of ischemia, infarction, and inflammatory lesions, while supporting monitoring of the therapeutic response. Lymphatic mapping with CEUS allows the identification of sentinel nodes and evaluation of nodal vascularity, enhancing staging and treatment planning in oncological settings. 3​

Cardiovascular applications include improved endocardial border delineation, myocardial perfusion assessment, evaluation of intracardiac masses or thrombi, shunt detection, and quantification of ventricular function. 16​ Vascular applications encompass dynamic evaluation of carotid plaque vulnerability, aortic endoleaks, peripheral perfusion, and venous disease, with quantitative perfusion parameters aiding assessment of vascular malformations and monitoring of the therapeutic response. 3​ 17​​ ​18​ In musculoskeletal imaging, CEUS enhances the detection of microvascularization under inflammatory conditions, such as active synovitis in rheumatoid arthritis, where it outperforms conventional Doppler ultrasound and provides real-time monitoring of treatment responses. 3​ ​19​ Pulmonary and pleuropulmonary applications include differentiation of inflammatory, ischemic, and malignant lesions, guiding interventions, and monitoring treatment responses in pneumonia, pleural effusions, pulmonary embolism, tumors, and COVID-19-related lung injury. 3​ ​20​ At the bedside, CEUS complements conventional point-of-care ultrasound by enabling the visualization of tissue microvascular perfusion, improving detection of infarction, infections, vascular abnormalities, and postoperative complications. 21​

CEUS also shows promise in maternal imaging as a radiation-free alternative to CT and MRI, allowing the accurate, real-time assessment of maternal pathologies without fetal exposure to ionizing radiation or placental-crossing CAs. 22​

Despite its broad utility , CEUS remains underutilized in Saudi Arabia, with a lack of reported evidence regarding its prescription and application in relevant clinical cases. 23​ Addressing this gap is essential, as a clear understanding of current practice can inform national planning, guide resource allocation, and define workforce training needs for competent CEUS use.

Documenting regional adoption and identifying barriers to adoption can inform training strategies, resource allocation, and integration into routine diagnostic workflows, ultimately optimizing patient outcomes and contributing to the international literature by showing how CEUS diffusion varies across healthcare systems and providing a reference for other emerging markets with similar constraints.

This cross-sectional survey therefore aims to examine the current CEUS use in Saudi Arabia and identifies barriers to wider adoption, providing an evidence base for strategies to strengthen clinical and research integration.

### Objectives

To assess current patterns of CEUS use in clinical and research settings in Saudi Arabia.
To identify key barriers limiting CEUS adoption.
To provide evidence-based strategies to enhance CEUS awareness, accessibility, and utilization.

## Methods

This cross-sectional, survey-based study employed a web-based questionnaire, developed by a principal investigator and hosted on SurveyMonkey and Qualtrics. It was distributed electronically to more than 5,000 potentially eligible individuals via email, the Saudi Commission for Health Specialties survey distribution service, and professional social media platforms (e.g., LinkedIn and WhatsApp) between July and October 2025. A target sample of 100 practitioners was deemed practical and sufficient to capture current usage patterns and perceived barriers, given the absence of publicly available national CEUS procedure counts. The survey was disseminated in three rounds to enhance participation, yielding 92 completed responses, highlighting challenges in recruiting CEUS practitioners within the local context. The survey targeted professionals currently working in Saudi Arabia who are engaged in medical imaging or ultrasound practice, including radiologists, sonologists, referring physicians, sonographers, researchers, and sales representatives of ultrasound equipment or CAs. Individuals not based in Saudi Arabia, students or interns, non-English speakers, and those not professionally involved in ultrasound practice, research, or sales were excluded.

A preliminary version of the instrument was reviewed by five ultrasound graduates to assess its clarity, flow, and usability, and their feedback informed the final survey design. The final questionnaire was administered in English and included items on demographic characteristics, current CEUS use and training, perceived barriers to adoption, and recommendations for improving implementation. The full survey is provided in Appendix A, and the cover page with an informed consent text is presented in the supplementary file.

Ethical approval was obtained from King Abdullah Bin Abdulaziz University Hospital, Institutional Review Board (Log No. 25-0116). The consent statement on the cover page indicated that participation was voluntary, and that submission of a completed survey implied informed consent. The survey was conducted anonymously, with no personal identifiers required. Participants had the option to provide email voluntarily for potential follow-up communication.

Data were cleaned and refined prior to analysis, including recoding free-text responses into standardized categories. Frequency-of-use items (e.g., liver lesion characterization) were coded on an ordinal scale from 0 to 4, where 0 = never, 1 = rarely, 2 = monthly, 3 = weekly, and 4 = daily.

### Statistical analysis

Categorical variables were summarized using frequencies and percentages. Continuous variables were reported as means with standard deviations or medians with interquartile ranges (IQRs), as appropriate based on data distribution. Qualitative responses were summarized descriptively rather than subjected to full thematic analysis due to limited responses. All statistical analyses and graphical presentations were performed using JMP student edition 18.2.1 (SAS Institute Inc., Cary, NC).

## Results

The survey yielded 92 completed responses following three distribution rounds, underscoring the difficulty of engaging CEUS practitioners within the current local landscape. Online dissemination enabled the survey to reach practitioners across multiple regions, demonstrating the practicality of digital recruitment in a geographically dispersed workforce.

### Participants' characteristics

Most respondents were aged 35–44 years (37%), with additional representation from the 45–54 (24%) and 25–34 (21%) groups. Only a minority were aged 55 years or older (19% in total). Overall, women represented 33% and men 67%. Median (IQR) years of experience were 13 (10–20) years. The geographic distribution of respondents is shown in Fig. 1 , with the majority located in the Central region of Saudi Arabia.

**Fig. 1:**
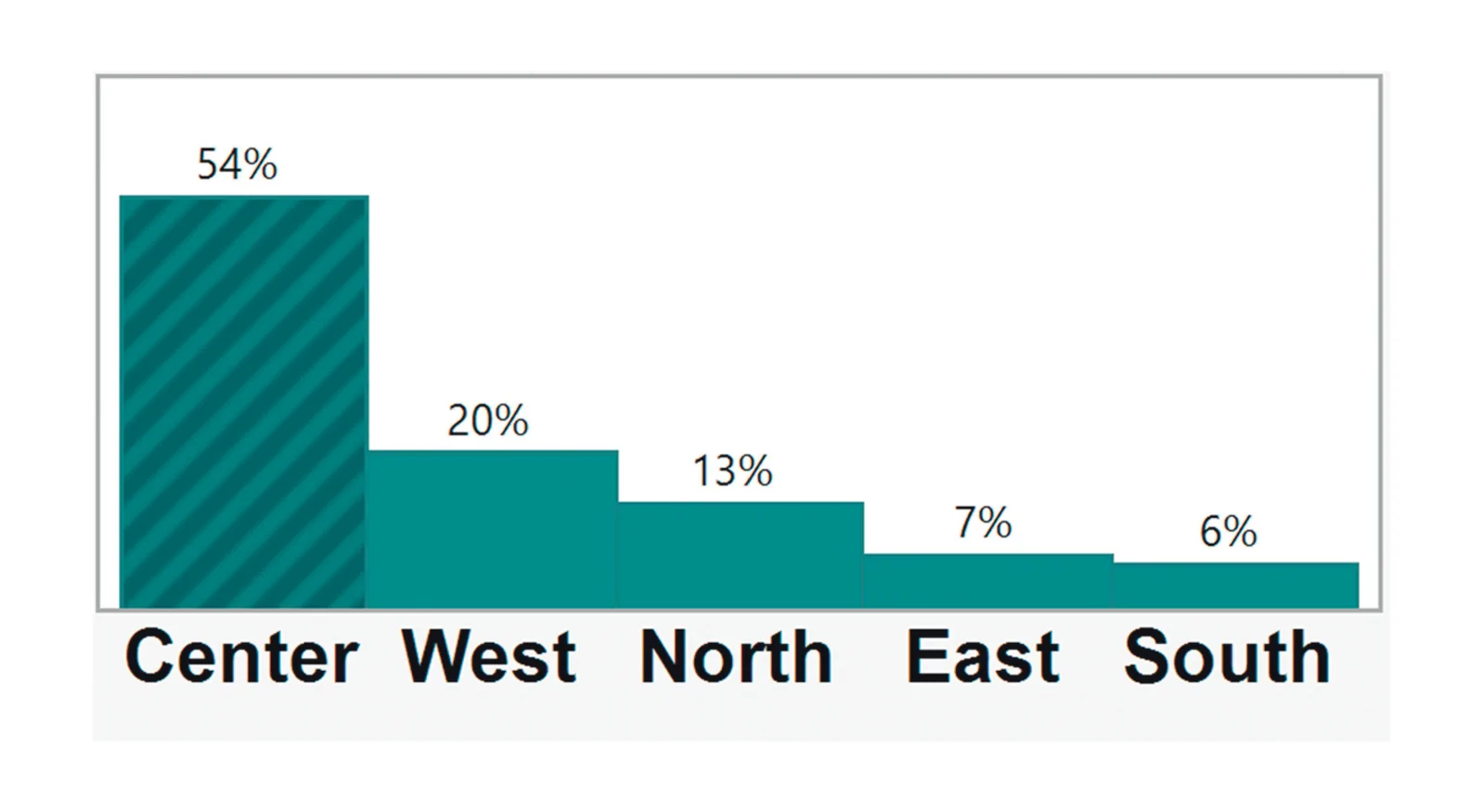
Geographical distribution of respondents across Saudi Arabia

Respondents represented various professional roles, led by referring physicians (35%) and radiologists (23%), followed by other roles (18%), sonographers (15%), board-certified sonologists (5%), ultrasound researchers (2%), and CEUS sales representatives (1%). Most respondents worked in hospitals (68%), with smaller proportions employed in private clinics (15%) and academic institutions (12%).

### Current use of contrast-enhanced ultrasound

CEUS use was limited, with the vast majority of respondents (79%) reporting no current clinical use. Of those who used it clinically, over half (55%, 11/20) reported performing only 1 to 5 CEUS examinations/ mo, while smaller proportions reported 6 to 10 examinations (30%, 6/20), 11 to 20 examinations (10%, 2/20), or more than 20 examinations/mo (5%, 1/20).

Among respondents who reported using CEUS clinically, just over half (55%; 11/20) performed 1 to 5 CEUS examination/mo. Smaller proportions reported 6 to 10 examinations (30%, 6/20), 11 to 20 examinations (10%, 2/20), or more than 20 examinations/mo (5%, 1/20). In total, 23% of respondents reported that their institution has an established CEUS protocol.

CEUS usage was generally low across most clinical indications ( Fig. 2 ). A small number of respondents provided additional free-text indications not listed in the survey. These included intra-arterial shunting, research applications, and thyroid imaging. The distribution of responses demonstrated that most indications were infrequently performed, with the majority clustered in the “never” or “rarely” categories and only a small proportion reflecting routine (weekly or daily) use.

**Fig. 2:**
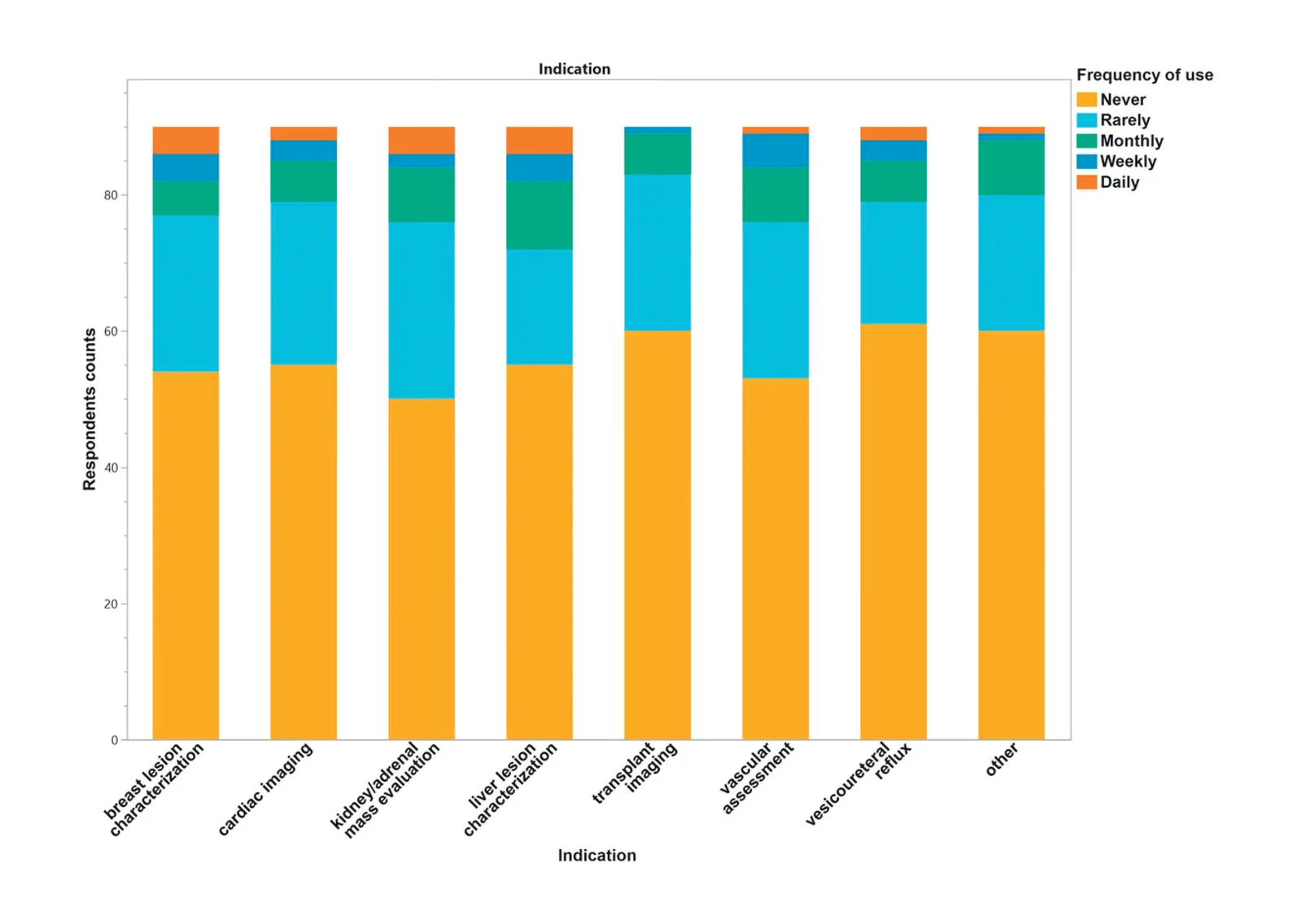
Distribution of CEUS usage frequency across clinical indications. Bars represent the number of respondents. Colors indicate the frequency of use on a five-point scale (never, rarely, monthly, weekly, and daily). Most indications were predominantly rated as “never” or “rarely” used, with limited routine (weekly or daily) implementation across clinical settings. A small number of respondents provided additional free-text indications not listed in the survey. These included intra-arterial shunting, research applications, and thyroid imaging.

Most respondents reported that they do not currently use CEUS in research (87%), with only a minority indicating active research use (13%). Of those, only three specified applications: liver study, renal study, and one involving pulmonary arteriovenous fistulas. General electric healthcare ultrasound systems were the most reported (17%), followed by Philips ultrasound systems (16%) and Samsung ultrasound systems (2%), with the remaining responses either unspecified models or non-applicable answers.

Awareness of CEUS clinical benefits varied among respondents. Nearly half of the respondents (48%) reported being somewhat aware, while 20% indicated they were extremely aware. In contrast, 19% reported being not so aware, and 13% reported that they were not at all aware.

The largest proportion reported being somewhat aware (48%), followed by extremely aware (20%). Nineteen percent reported being not so aware, and 13% reported that they were not at all aware. Most respondents had not received formal CEUS training, although many expressed interest in obtaining it ( Fig. 3 ). Among those who had received CEUS training, the most common training setting was on-the-job clinical training ( Fig. 4 ).

**Fig. 3:**
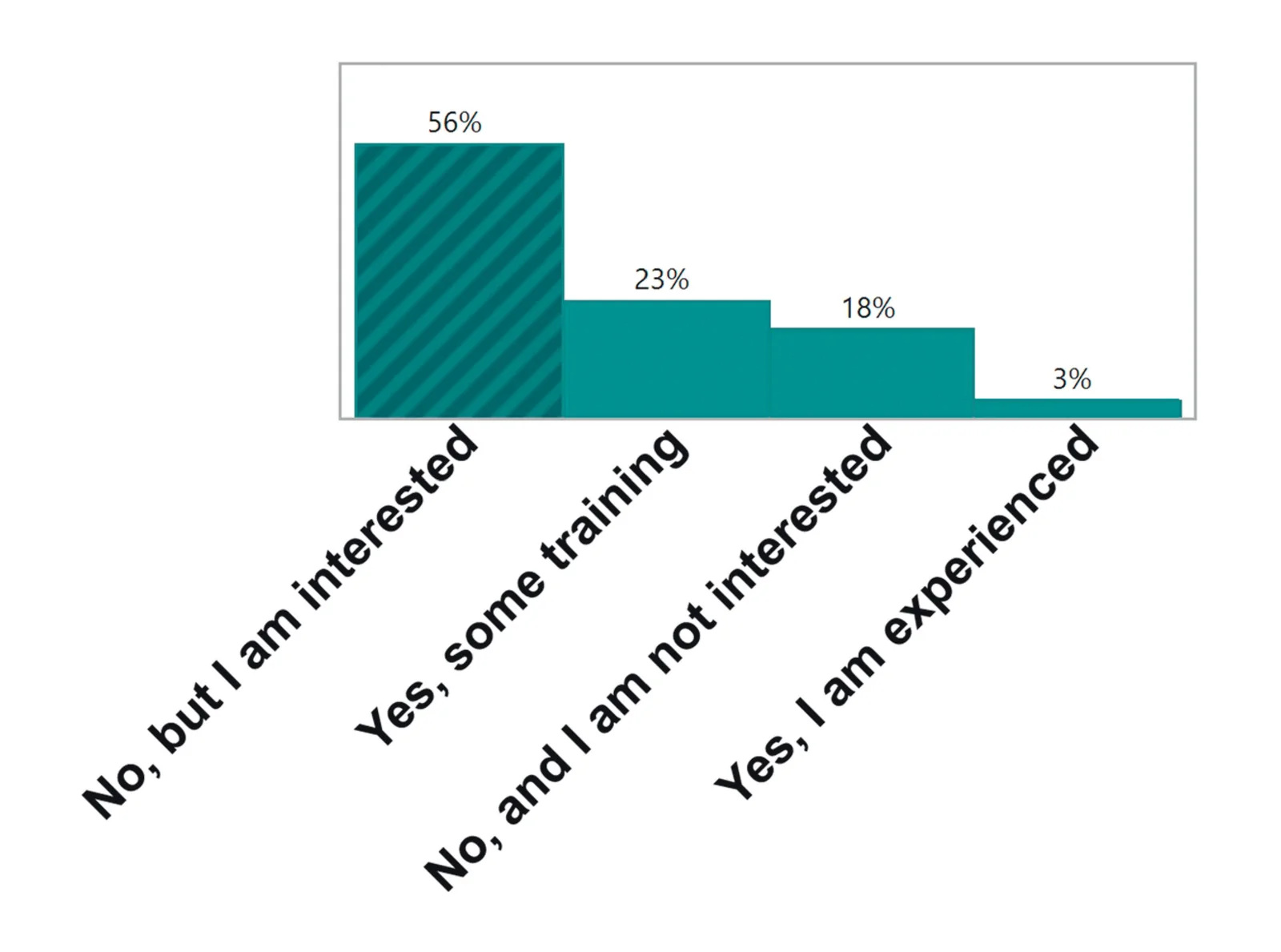
Level of CEUS training among respondents.

**Fig. 4:**
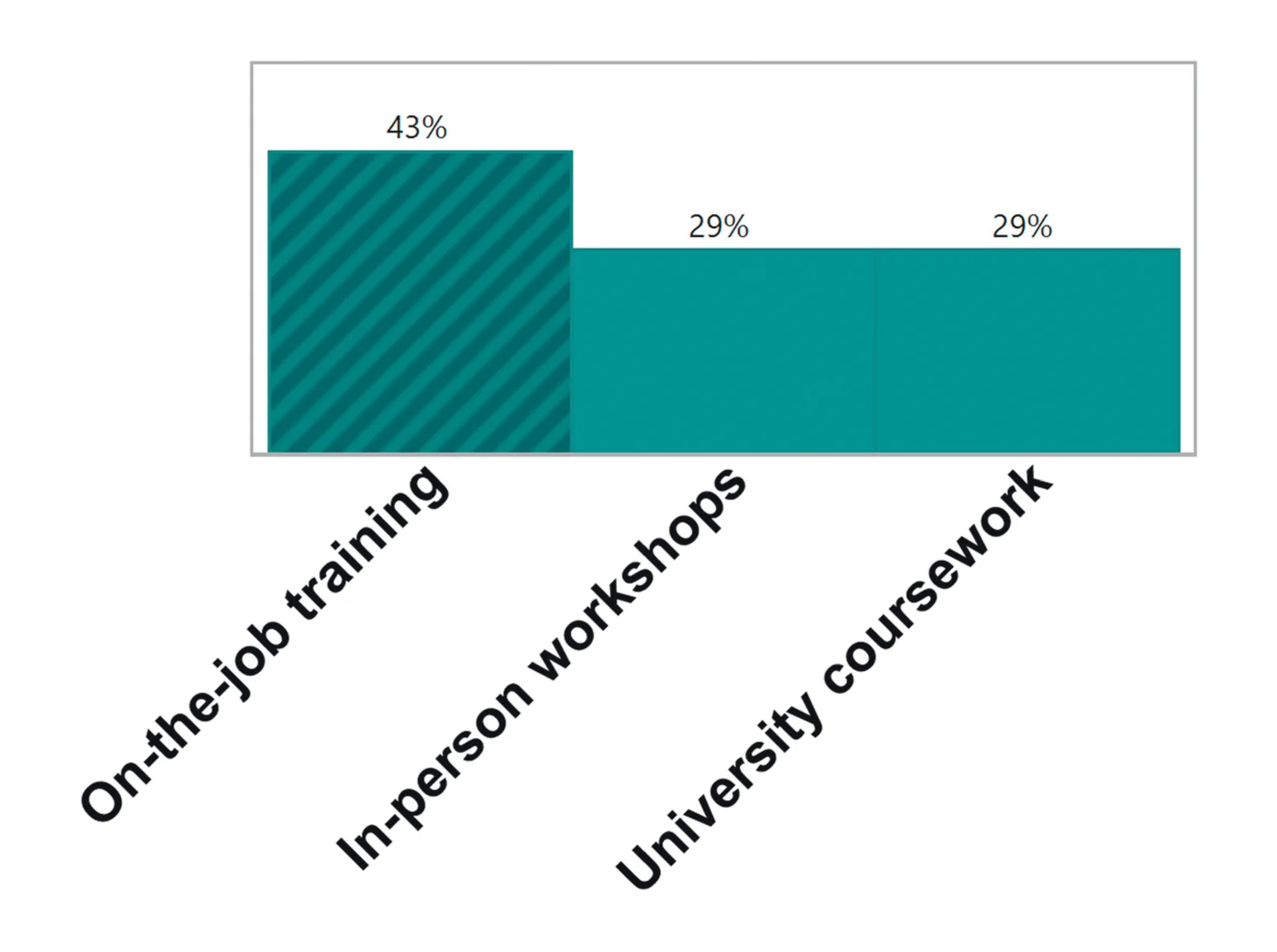
Reported settings in which respondents received CEUS training.

### Barriers to wider contrast-enhanced ultrasound adoption

Fig. 5 represents respondents' barriers to CEUS implementation where stacked bars represent the number of respondents selecting each barrier severity level on a six-point scale (not applicable, not a barrier, minor barrier, moderate barrier, substantial barrier, and major barrier). Also, 30% of respondents reported that CEUS-capable equipment is available in their institution.

**Fig. 5:**
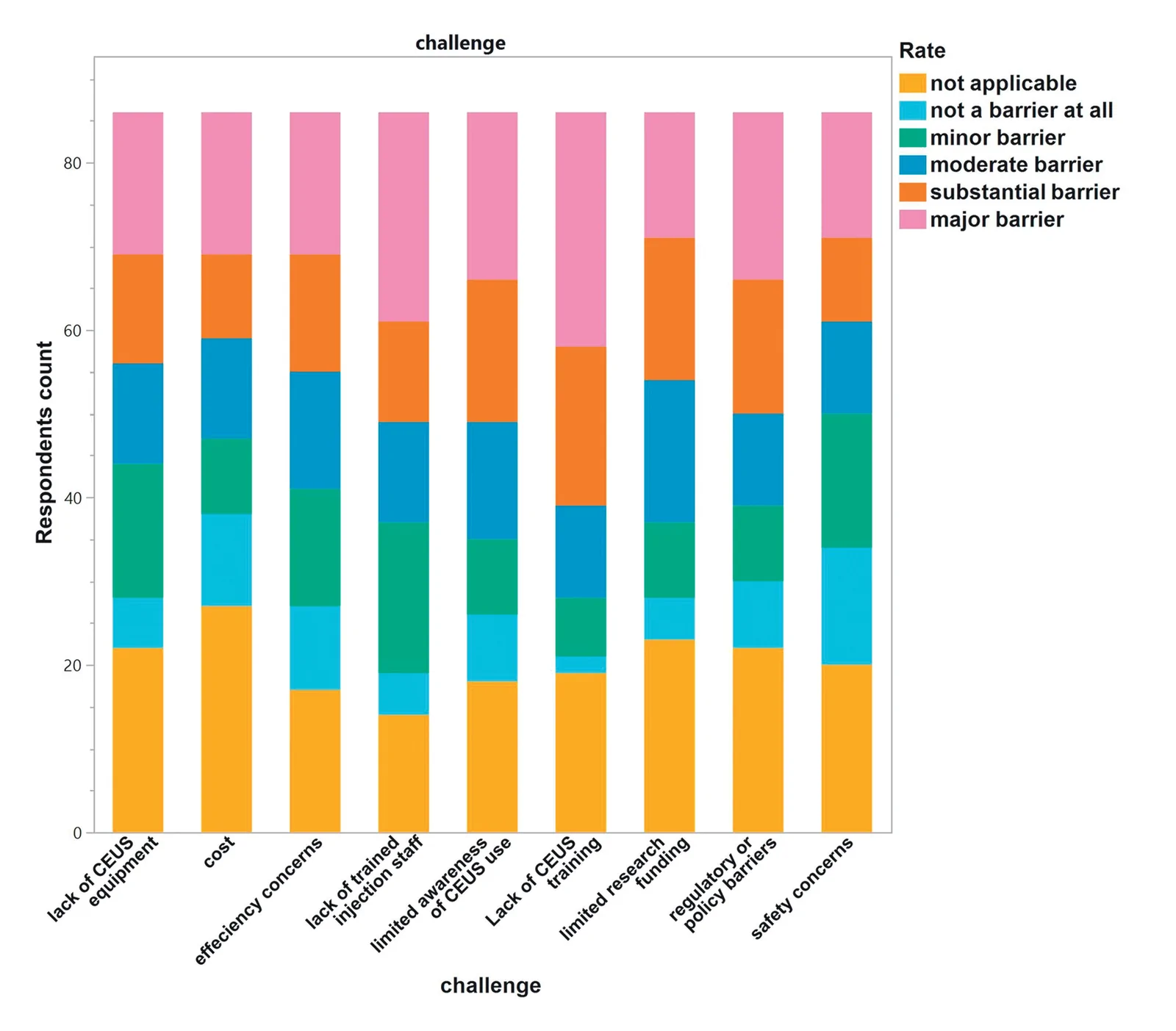
Perceived barriers to CEUS adoption across participating institutions. Stacked bars represent the number of respondents selecting each barrier severity level on a six-point scale.

Regarding the support required from CEUS sales representatives to facilitate research collaboration, 31% of respondents indicated a need for training and educational support, while 28% reported requiring financial sponsorship ( Fig. 6 ).

**Fig. 6:**
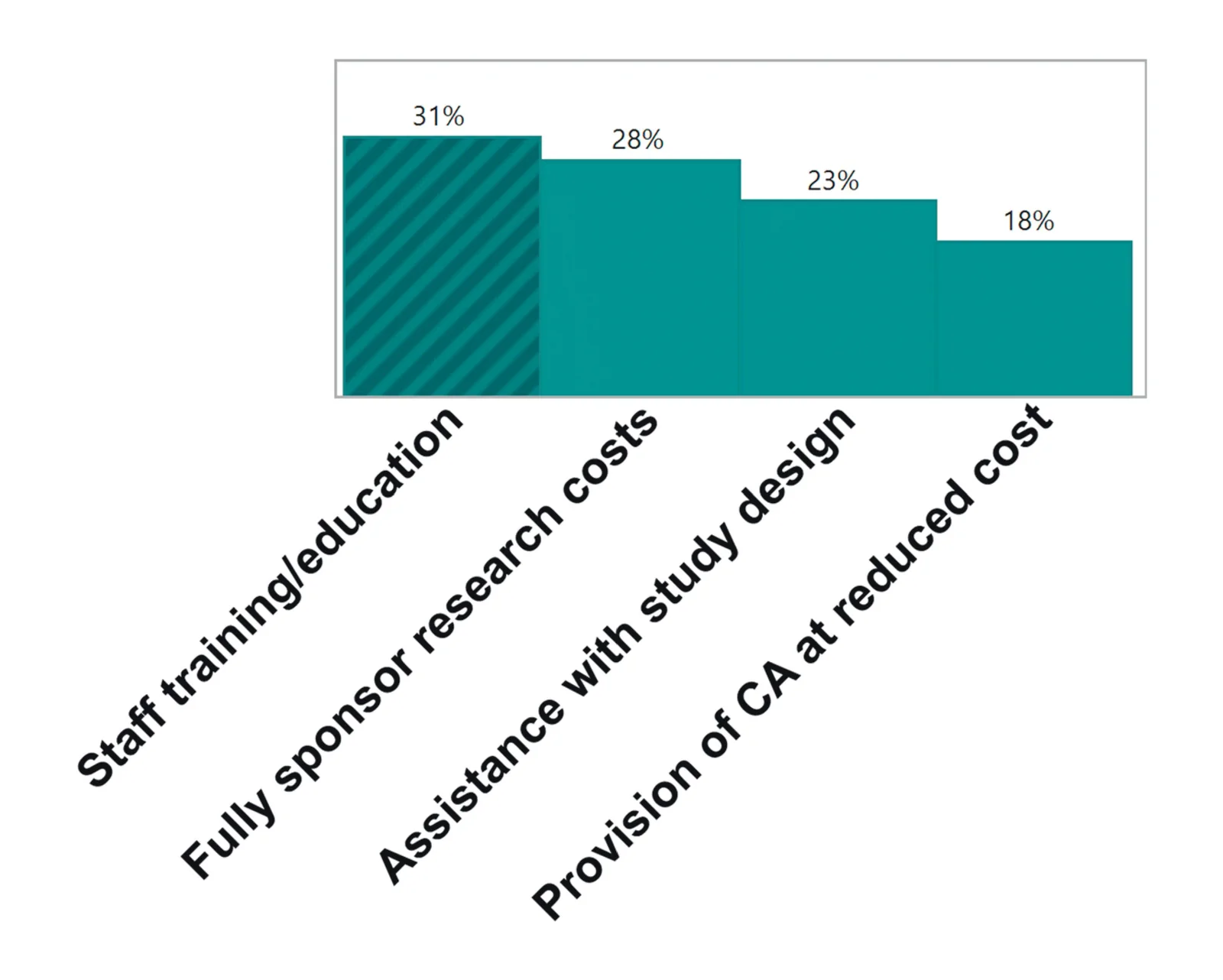
Forms of support needed from CEUS suppliers to facilitate research collaboration.

One ultrasound equipment representative and two CA distributors completed the sales representative questions and reported challenges in promoting CEUS to healthcare providers included insufficient understanding of CEUS benefits (selected twice), competition from other imaging modalities, pricing concerns, and regulatory restrictions (each selected once). Finally, in response to the item on factors that could improve CEUS adoption in the market, the representatives selected increased training and education, with collaborative research also identified twice as a key facilitator of broader uptake.

For the optional open-ended items related to sales representatives or supplier collaboration in CEUS research, respondents ( *n* = 3) highlighted the need to expand CEUS availability within polyclinic settings, increase access to structured training opportunities, and strengthen direct engagement between suppliers and end users.

## Discussion

This survey demonstrates limited CEUS utilization in Saudi Arabia despite adequate regional representation and an experienced ultrasound workforce. Only one-fifth of respondents reported any clinical use and an even smaller proportion performed CEUS at least monthly, reflecting a substantial gap between technological availability and actual practice. Although ultrasound CAs are approved in more than 70 countries for cardiac, hepatic, gastrointestinal, renal, and vascular applications, local uptake remains modest. 24​

When examining the major barriers, lack of CEUS training and insufficient trained staff to perform injections emerged as the most prominent obstacles. Regulatory and policy barriers followed, alongside the limited awareness of CEUS applications and inadequate CEUS-capable equipment. These findings are consistent with the international literature showing that CEUS remains underutilized despite evidence-based guidelines supporting its use, largely due to inconsistencies in sonographer training and challenges related to establishing IV access and administering ultrasound CAs.

The identification of inadequate injection-training capacity is particularly noteworthy because established international guidelines already support sonographer-led contrast administration. The American Society of Echocardiography (ASE) endorses sonographer-driven decision-making for contrast use and IV access by trained practitioners, 25​ and International Contrast Ultrasound Society (ICUS) similarly encourages scope-of-practice policies enabling qualified Diagnostic Medical or Cardiac Sonographers to obtain IV access when medically indicated. The persistence of this barrier suggests that such competency-based pathways are not yet widely implemented locally, emphasizing the need for clearer institutional policies and structured training programs.

These findings parallel those of the ICUS–ASE collaborative survey, 26​ which documented substantial gaps in CEUS education across U.S. sonography programs. Only 54% included the CEUS content, and IV-insertion training was offered in only 18%. Similarly, in our study, the lack of CEUS training was the strongest barrier, followed by the shortage of trained injection staff. The high Saudi interest in CEUS training (56%) indicates that motivation is not the limiting factor; structural and institutional issues appear to drive the deficit.

This strong demand for foundational training is notable given CEUS’s global diffusion and the presence of organizations dedicated to CEUS education and guideline development—including ICUS, EFSUMB, and WFUMB. We found that most respondents gained CEUS exposure through on-the-job learning, which may contribute to inconsistent practice standards. Together, these findings reinforce the need for more accessible local training opportunities, clearer awareness of existing international resources, and collaborative initiatives to bridge knowledge and practice gaps.

In this context, educational initiatives—through local workshops, industry collaboration, or adaptation of international curriculum modules—represent practical approaches to developing a competent workforce. More explicit regulatory and operational guidance may also reduce clinician uncertainty and facilitate broader adoption across established clinical indications and ethically governed research environments.

Comparable system-level challenges have been documented internationally. O’Brien et al reported limited CEUS adoption within the UK National Health Service due to low demand, constrained referral pathways, and insufficient practitioner training. 27​ Their analysis showed that expanding the CEUS use could generate substantial cost savings, reduce wait times, and increase diagnostic capacity. The convergence between these findings and the present results underscores the broader relevance of improving CEUS awareness, training, and regulatory clarity as part of a sustainable imaging strategy.

Financial barriers also warrant attention. Costs related to CEUS materials and limited research funding could be alleviated through sponsorship pathways and stronger industry engagement, including subsidized access to CAs and training resources. Such mechanisms would reduce the financial burden on healthcare institutions and support broader CEUS adoption.

Patterns of CEUS use among active users revealed that monthly use dominated, particularly for abdominal indications such as kidney/adrenal and liver lesion evaluation, followed by vascular and, to a lesser extent, cardiac applications. The limited cardiac use was unexpected given the CEUS’s established value in echocardiography for improving diagnostic performance and reader confidence. 1​

Supplier and distributor perspectives, although limited, emphasized insufficient clinician understanding of CEUS benefits and highlighted opportunities for greater training, closer vendor–clinician engagement, and collaborative research. These recommendations align closely with needs identified by respondents, reinforcing the potential value of coordinated efforts across institutions, suppliers, societies, and regulators.

## Conclusions

This study demonstrates that CEUS remains underutilized in Saudi Arabia despite an experienced ultrasound workforce and broad regional representation. Key barriers include limited training opportunities, regulatory uncertainty, and insufficient institutional readiness. Addressing these gaps is essential for integrating CEUS more effectively into routine clinical pathways. Immediate efforts should focus on expanding structured training, clarifying regulatory guidance, and improving institutional support to enhance clinical adoption and research engagement.

## Supplementary Material

Supplementary Material Zusatzmaterial
