# Supplementary material for: Barriers to the Broader Use of Contrast-Enhanced Ultrasound in Saudi Healthcare and Medical Research
Source: Ultrasound Int Open. 2026 Jul 13;12:a28990967. doi: 10.1055/a-2899-0967 (PMC13643735; doi:10.1055/a-2899-0967)
Supplement: Supplementary file 1 [file 10-1055-a-2899-0967_29036044.pdf]

Section 1: Demographic Information

Age range

- 18 to 24
- 25 to 34
- 35 to 44
- 45 to 54
- 55 to 64
- 65 to 74
- 75 or older

Gender

- Male
- Female

Region/City in Saudi Arabia ------------------

Position/Role:

- Radiologist
- Referring physician
- Sonologist
- Sonographer
- Ultrasound or CEUS sales Representative
- Researcher with experience in ultrasound imaging
- Other (please specify): ___________

Institution/Workplace:

- Hospital
- University or college
- Research center or institute

Ultrasound International Open | DOI 10.1055/a-2899-0967 | © The Author(s).

- Private clinic
- Imaging center
- Ultrasound or Contrast agent center
- Other (please specify) __________

Years of experience: __________

Section 2: Current Use of CEUS

Do you currently use CEUS in your 'clinical' practice (not research) at your institution?

- Yes
- No

Approximately how many CEUS examinations are performed per month at your institution?

- None
- 1–5
- 6–10
- 11–20
- More than 20
- Not sure

Is there a dedicated CEUS protocol/guidelines for performing CEUS exams at your institution?

Ultrasound International Open | DOI 10.1055/a-2899-0967 | © The Author(s).

- Yes
- No
- Not applicable

For each of the following clinical indications, please indicate how often you use CEUS:
(Select one per row)

Clinical Indication	Daily	Weekly	Monthly	Rarely	Never
Liver lesion characterization	☐	☐	☐	☐	☐
Kidney/adrenal mass evaluation	☐	☐	☐	☐	☐
Breast lesion characterization	☐	☐	☐	☐	☐
Cardiac imaging	☐	☐	☐	☐	☐
Vascular assessment	☐	☐	☐	☐	☐
Transplant imaging Vesicoureteral reflux	☐	☐	☐	☐	☐
Other (please specify): _____	☐	☐	☐	☐	☐

Have you used CEUS in research?

- Yes
- No

If yes, what clinical application or body region was it used for in research? *(Short answer)*

____

Which ultrasound system(s) have you used for CEUS, either clinically or in research?
(Please specify the company name and model, e.g., GE LOGIQ E9, Philips EPIQ 7)

How aware are you of the benefits of using CEUS in clinical practice?

- Very aware
- Somewhat aware

Ultrasound International Open | DOI 10.1055/a-2899-0967 | © The Author(s).

- Somewhat unaware (not so aware)
- Very unaware (not at all aware)

Have you received any formal training related to CEUS?

- Yes, I am experienced
- Yes, some training
- No, I am interested
- No, and I am not interested

If you have received training, please specify the type: *(Select all that apply)*

- University coursework
- In-person workshops
- Online courses or webinars
- On-the-job training
- Other (please specify): ___________

Section 3: Barriers to wider CEUS adoption

What do you perceive as the main barriers to the wider adoption of CEUS in Saudi Arabia?
(Please rate each item from 1 = Not a barrier at all to 5 = Major barrier)

Barrier	1	2	3	4	5
Lack of awareness about its application	☐	☐	☐	☐	☐
Limited training opportunities	☐	☐	☐	☐	☐
High costs	☐	☐	☐	☐	☐
Lack or insufficient research funding	☐	☐	☐	☐	☐
Regulatory or policy barriers	☐	☐	☐	☐	☐
Concerns about safety	☐	☐	☐	☐	☐

Ultrasound International Open | DOI 10.1055/a-2899-0967 | © The Author(s).

Concerns about efficiency	☐	☐	☐	☐	☐
Lack of equipment with CEUS software	☐	☐	☐	☐	☐
Need for additional qualified staff to perform injections	☐	☐	☐	☐	☐

Other (please specify): ___________

Does your institution currently have an ultrasound machine with CEUS capability?

- Yes
- No
- Not sure

What support would you need from CEUS sales representatives to facilitate research collaboration? *(Select all that apply)*

- Fully sponsor your research costs
- Provision of Sonovue to the institution at reduced cost
- Access to staff training/education
- Assistance with study design/methodology
- Other (please specify): ___________

Additional comments or suggestions regarding sales representative/ supplier collaboration for CEUS research (optional). ___________

Section 4: Sales Representative Section *(To be completed only if you are a sales representative)*

Are you a sales representative involved in CEUS-related products?

- Yes – **Contrast agent distributor**

Ultrasound International Open | DOI 10.1055/a-2899-0967 | © The Author(s).

- Yes – **Ultrasound equipment representative**
- No *(If selected, skip this section)*

What challenges do you face in promoting CEUS to healthcare providers? *(Select all that apply)*

- Lack of interest from providers
- Insufficient understanding of CEUS benefits
- Competition from other modalities
- Pricing concerns
- Regulatory restrictions
- Other (please specify): ___________

What do you believe would improve CEUS adoption in the market? *(Select all that apply)*

- Increased training and education
- Collaborative research
- Improved reimbursement policies
- Targeted marketing campaigns
- Other (please specify): ___________

Additional comments or suggestions from local CEUS sales representative/ supplier? (optional)

Section 5: Contact Information (optional)

This will not be reported in the study and will only be used for potential follow-up or collaboration.

Ultrasound International Open | DOI 10.1055/a-2899-0967 | © The Author(s).

Email: ____________

Thank you for your time!

Ultrasound International Open | DOI 10.1055/a-2899-0967 | © The Author(s).
